# BDNF/BDNF-AS Gene Polymorphisms Modulate Treatment Response and Remission in Bipolar Disorder: A Randomized Clinical Trial

**DOI:** 10.3390/jpm15020062

**Published:** 2025-02-07

**Authors:** Anton Shkundin, Heather E. Wheeler, James Sinacore, Angelos Halaris

**Affiliations:** 1 Department of Psychiatry and Behavioral Neurosciences, Loyola University Chicago, Maywood, IL 60153, USA; 2 Department of Biology, Loyola University Chicago, Chicago, IL 60660, USA; 3 Parkinson School of Health Sciences and Public Health, Loyola University Chicago, Maywood, IL 60153, USA

**Keywords:** bipolar disorder, treatment-resistant depression, brain-derived neurotrophic factor (BDNF), selective serotonin reuptake inhibitors (SSRIs), cyclooxygenase-2 (COX-2), escitalopram, celecoxib, rs1519480, rs6265, rs10835210

## Abstract

**Background:** Bipolar disorder (BD) is a chronic condition associated with treatment resistance, cognitive decline, structural brain changes, and an approximately 13-year reduction in life expectancy compared to the general population. Depression in BD substantially impairs quality of life, while neuroinflammation and excitotoxicity are thought to contribute to the recurrence of mood episodes and disease progression. Brain-derived neurotrophic factor (BDNF) plays a key role in neuronal growth and function, with its dysregulation being linked to various psychiatric disorders. This study is an extension of a previously published clinical trial and was conducted to assess the effects of three BDNF and BDNF-AS gene polymorphisms (rs1519480, rs6265, and rs10835210) on treatment outcomes and serum BDNF levels in patients with treatment-resistant bipolar disorder depression (TRBDD) over an eight-week period. **Methods:** This study included 41 participants from a previously conducted randomized clinical trial, all of whom had available BDNF serum samples and genotype data. The participants, aged 21 to 65, were diagnosed with bipolar disorder, and treatment-resistant depression was assessed using the Maudsley Staging Method. Participants were randomly assigned to receive either escitalopram plus a placebo (ESC+PBO) or escitalopram plus celecoxib (ESC+CBX) over an 8-week period. Statistical analyses included a mixed ANOVA and chi-square tests to compare the minor allele carrier status of three SNPs with treatment response and remission rates. **Results:** Non-carriers of the rs6265 A allele (*p* = 0.005) and carriers of the rs10835210 A allele (*p* = 0.007) showed a significantly higher response to treatment with adjunctive celecoxib compared to escitalopram alone. Additionally, remission rates after adjunctive celecoxib were significantly higher in both carriers and non-carriers across all three SNPs compared to escitalopram alone. However, remission rates were notably higher in non-carriers of the rs1519480 G allele and rs10835210 A allele, as well as in carriers of the rs6265 A allele. **Conclusions:** This study suggests that genetic variations in BDNF and BDNF-AS genes significantly influence treatment response to and remission with escitalopram and celecoxib in bipolar disorder.

## 1. Introduction

Bipolar disorder (BD) is a lifelong chronic illness often with a high rate of treatment resistance and potentially gradual cognitive decline and structural brain changes [1,2]. Neuroinflammation, associated with excitotoxicity and apoptosis, may contribute to both the recurrence of episodes and disease progression [2]. Among individuals with BD, life expectancy is approximately 13 years shorter than that of the general population [3]. Additionally, depression profoundly affects quality of life in those living with BD [4].

Brain-derived neurotrophic factor (BDNF) plays a crucial role in neuronal growth, differentiation, survival, and synaptic plasticity, and its dysregulation is implicated in the pathogenesis of various psychiatric disorders [5]. Antidepressants, such as escitalopram, have been shown to either restore BDNF function or prevent a decline in BDNF levels [6]. Escitalopram has also been found to modulate BDNF mRNA gene expression [7], with studies reporting a concentration-dependent increase in activity-dependent BDNF promoter IV activation following escitalopram exposure [8]. However, the impact of antidepressants on BDNF levels can vary widely, influenced by genetic factors, baseline BDNF concentrations, and the specific antidepressant used [9,10]. For instance, the BDNF Val66Met polymorphism (rs6265), a common genetic variant, has been shown to affect BDNF secretion and is associated with varying responses to SSRIs [11]. This variant can influence binding affinities or the stability of the BDNF protein, thereby altering its overall function [12].

Patients presenting with inflammation are likely to exhibit resistance to SSRIs, including escitalopram, as its chemical efficacy diminishes under inflammatory conditions [13]. Elevated levels of inflammatory markers before treatment are associated with poor antidepressant response [14]. Increasing evidence suggests that inflammatory processes play a significant role in the development of depression [15]. Inflammatory responses in peripheral tissues may trigger neuroinflammation in the brain, involving microglia, astrocytes, and oligodendrocytes, which contribute to the pathophysiology of depression [16,17]. Individual physiological differences influence the impact of inflammation, with some individuals being more susceptible to inflammation-related depression than others [18].

The overexpression of cyclooxygenase (COX), particularly COX-2, can result from excessive neuronal stimulation, potentially leading to progressive neuronal death in the hippocampus due to the overproduction of prostaglandins [19]. COX-2 upregulation has been linked to brain ischemia, as well as neurodegenerative and neuropsychiatric disorders, including major depressive disorder, bipolar disorder, and schizophrenia [20,21].

Adjunctive anti-inflammatory treatments are emerging as promising therapeutic approaches for managing unipolar and bipolar disorders, targeting immune–inflammatory dysfunction as a viable therapeutic component [22,23,24,25]. The inhibition of COX-2 expression reduces the release of inflammatory factors and oxidative stress while modulating the homeostasis of the monoamine transmitter system [26]. Selective COX-2 inhibitors, such as celecoxib, may inhibit microglial activation and glutamate release, while enhancing serotonergic and noradrenergic output in the prefrontal cortex [21]. Additionally, these inhibitors demonstrate antioxidant, neuroprotective, anti-inflammatory, and immunomodulatory properties [27,28]. They have shown effectiveness as adjunctive therapies in reducing depressive symptoms in both unipolar and bipolar depression [29,30,31,32,33].

Treatment resistance affects 20–60% of patients with psychiatric disorders and is linked to healthcare costs up to ten times higher than those of other patients [34]. At least 30% of individuals with depression meet the definition of treatment-resistant depression (TRD), characterized by an inadequate response to at least two antidepressants despite adequate treatment trials and adherence [35]. TRD is associated with chronic depression, suicidal behaviors, and impaired quality of life, with approximately 8% of its risk attributed to common genetic variation in unrelated individuals [36].

This study extends a previously published randomized clinical trial [1]. The primary aim of the present study was to evaluate the influence of three BDNF/BDNF-AS gene polymorphisms (rs1519480, rs6265, and rs10835210) on treatment outcomes and serum BDNF levels in patients with treatment-resistant bipolar disorder depression (TRBDD) over eight weeks of treatment.

## 2. Materials and Methods

This study included 41 participants from a randomized clinical trial (NCT01479829) [1], all of whom had available BDNF serum samples and genotype data. The participants were patients aged 21 to 65 years experiencing TRBDD. Treatment resistance was assessed using the Maudsley Staging Method. Exclusion criteria included comorbid psychiatric diagnoses (with the exception of anxiety disorders), abnormal findings in routine laboratory tests, known allergies or hypersensitivity to the study medications, and the use of concomitant medications contraindicated with the study treatments. Participants were randomly assigned to receive either escitalopram with a placebo (ESC+PBO) or escitalopram with celecoxib (ESC+CBX) over an 8-week period. BDNF serum levels were measured using the enzyme-linked immunosorbent assay (ELISA). Blood cell DNA was isolated, and genome-wide genotyping was carried out with the Infinium Multi-Ethnic Global-8 v1.0 Kit. Three single-nucleotide polymorphisms (SNPs)—rs1519480, rs6265, and rs10835210—were selected based on their known associations with affective disorders [37,38,39,40]. Carriers were defined as individuals possessing at least one minor allele for each SNP (rs1519480G, rs6265A, and rs10835210A). Statistical analyses involved a series of chi-square tests to evaluate the effects of BDNF/BDNF-AS SNP carrier status between responders and non-responders, and remitters and non-remitters, receiving either ESC+PBO or ESC+CBX. Further analysis using mixed ANOVA was performed to evaluate the interaction effects of time (Week 4 and Week 8) and adjusted mean BDNF levels based on BDNF SNP carrier status.

## 3. Results

### 3.1. rs1519480

**Figure 1:**
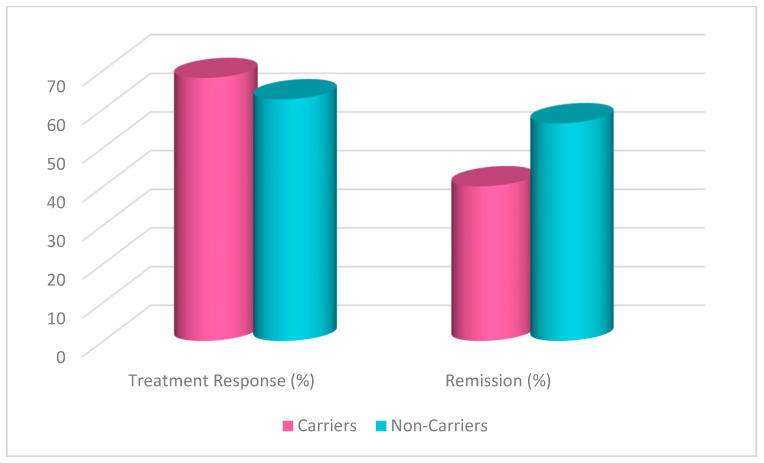
Effects of rs1519480 G carrier status on treatment response and remission.

A chi-square test was conducted to evaluate the effects of rs1519480 G carrier status on treatment response and remission (Figure 1) at Week 8. The cohort included non-carriers (n = 16) and carriers (n = 25). Non-carriers had a treatment response rate of 62.5%, while carriers had a response rate of 68.0%. The difference between the groups was not statistically significant (χ^2^(1) = 0.131, *p* = 0.717). Moreover, non-carriers had a remission rate of 56.3%, while carriers had a rate of 40.0%. The difference between the groups was not statistically significant (χ^2^(1) = 1.036, *p* = 0.309).

**Figure 2:**
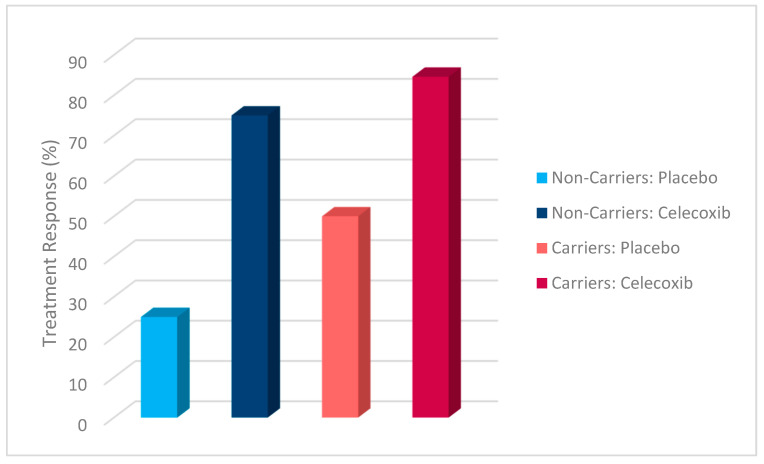
Effects of rs1519480 G carrier status and treatment group on treatment response.

**Figure 3:**
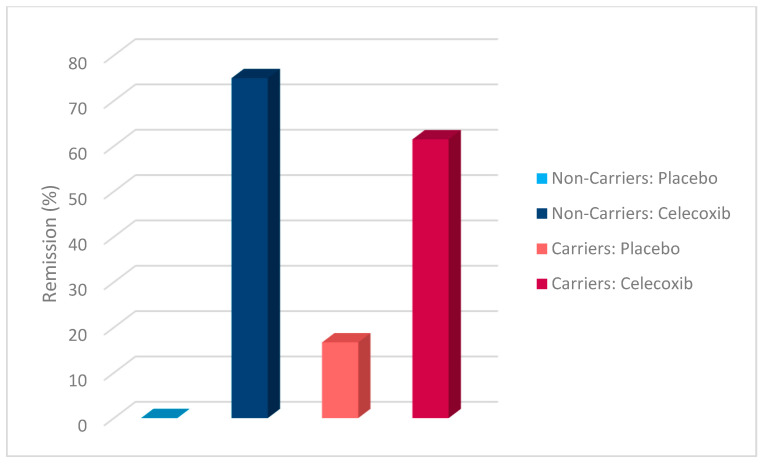
Effects of rs1519480 G carrier status and treatment group on remission.

Additionally, a chi-square test was conducted to evaluate the effects of rs1519480 G carrier status and treatment group on treatment response (Figure 2) and remission (Figure 3) at Week 8. The cohort included non-carriers (n = 16) and carriers (n = 25). Among non-carriers, 25% of the placebo group and 75% of the celecoxib group responded to treatment. For carriers, 50% of the placebo group and 84.6% of the celecoxib group had a treatment response. The difference between the groups did not reach statistical significance (χ^2^(1) = 3.200, *p* = 0.074 for non-carriers; χ^2^(1) = 3.436, *p* = 0.064 for carriers).

The effects of rs1519480 G carrier status and treatment group on remission rates demonstrated significant findings. Among non-carriers, 75% of the celecoxib group and 0% of the placebo group experienced remission. For carriers, 16.7% of the placebo group and 61.5% of the celecoxib group experienced remission. The difference between the groups was statistically significant for non-carriers (χ^2^(1) = 6.857, *p* = 0.009) and for carriers (χ^2^(1) = 5.235, *p* = 0.022).

**Figure 4:**
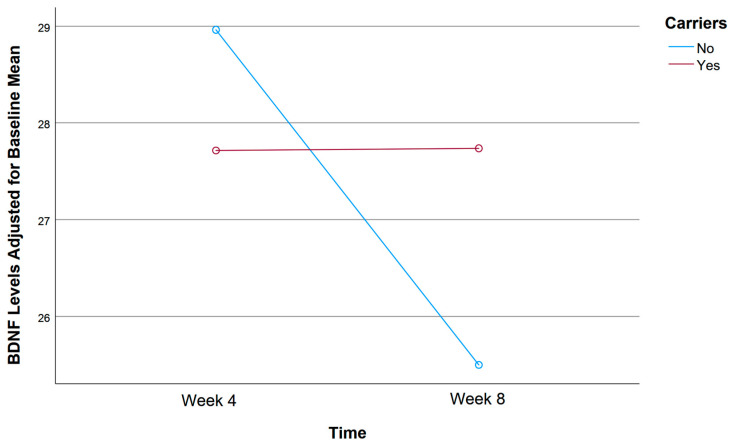
Effects of rs1519480 G carrier status and time (Week 4 and Week 8) on BDNF levels.

A mixed ANOVA was conducted to evaluate the effects of rs1519480 G carrier status and time on the adjusted mean BDNF levels from Week 4 to Week 8 (Figure 4). The entire cohort included non-carriers (N = 16) and carriers (N = 19) who had BDNF serum samples available at both follow-up time points. Non-carriers had an adjusted mean BDNF Wk4 of 28.96 and BDNF Wk8 of 25.50, showing a decline in BDNF levels from Week 4 to Week 8. In contrast, carriers had an adjusted mean BDNF Wk4 of 27.72 and BDNF Wk8 of 27.74, showing a relatively stable BDNF level with minimal change over the same period. Despite these notable trends, the difference between the groups did not reach statistical significance (F(1,32) = 1.041, *p* = 0.315).

### 3.2. rs6265

**Figure 5:**
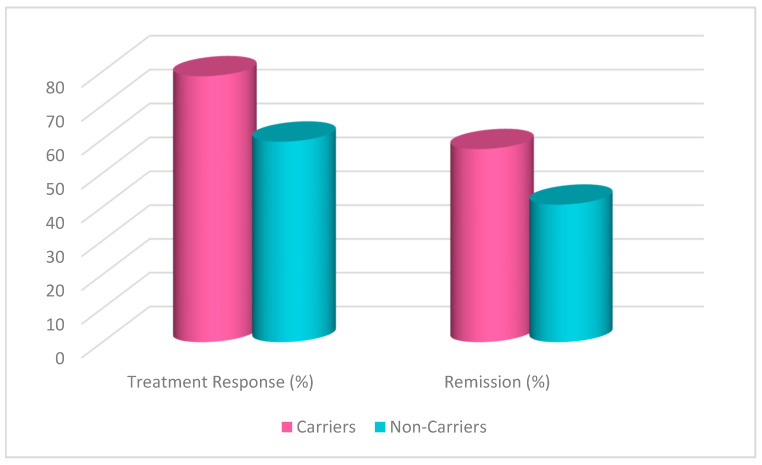
Effects of rs6265 A carrier status on treatment response and remission.

A chi-square test was conducted to evaluate the effects of rs6265 A carrier status on treatment response and remission (Figure 5) at Week 8. The cohort included non-carriers (n = 27) and carriers (n = 14). Non-carriers had a treatment response rate of 59.3%, while carriers had a response rate of 78.6%. The difference between the groups was not statistically significant (χ^2^(1) = 1.529, *p* = 0.216). Moreover, non-carriers had a remission rate of 40.7%, while carriers had a rate of 57.1%. The difference between the groups was not statistically significant (χ^2^(1) = 0.997, *p* = 0.318).

**Figure 6:**
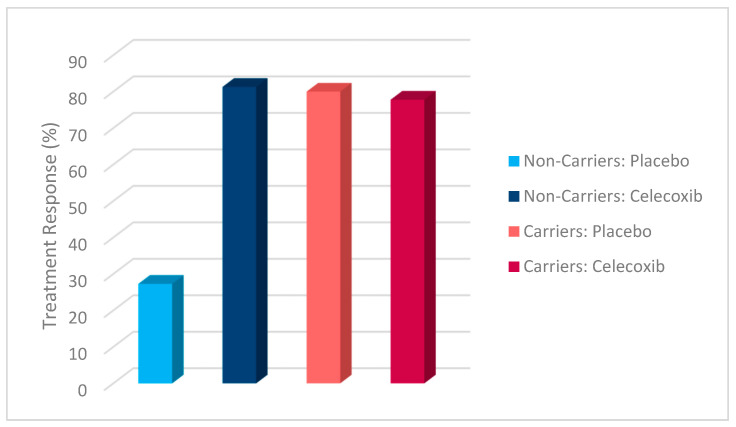
Effects of rs6265 A carrier status and treatment group on treatment response.

**Figure 7:**
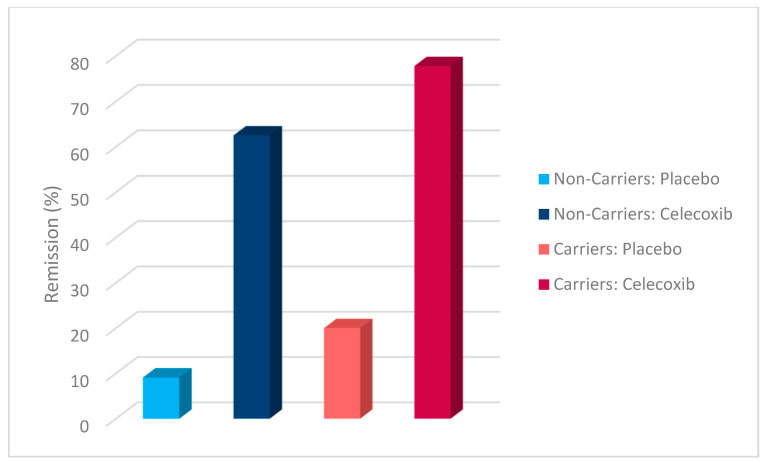
Effects of rs6265 A carrier status and treatment group on remission.

Additionally, a chi-square test was conducted to evaluate the effects of rs6265 A carrier status and treatment group on treatment response (Figure 6) and remission (Figure 7). The cohort included non-carriers (n = 27) and carriers (n = 14). Among non-carriers, 27.3% of the placebo group and 81.3% of the celecoxib group responded to treatment. For carriers, 80% of the placebo group and 77.8% of the celecoxib group responded. The difference between the groups was statistically significant for non-carriers (χ^2^(1) = 7.867, *p* = 0.005) but not for carriers (χ^2^(1) = 0.009, *p* = 0.923).

Furthermore, the effects of rs6265 A carrier status and treatment group on remission rates demonstrated significant findings. Among non-carriers, 9.1% of the placebo group and 62.5% of the celecoxib group experienced remission. In contrast, carriers demonstrated a remission rate of 20% in the placebo group but 77.8% in the celecoxib group. The difference between the groups was statistically significant for non-carriers (χ^2^(1) = 7.702, *p* = 0.006) and for carriers (χ^2^(1) = 4.381, *p* = 0.036).

**Figure 8:**
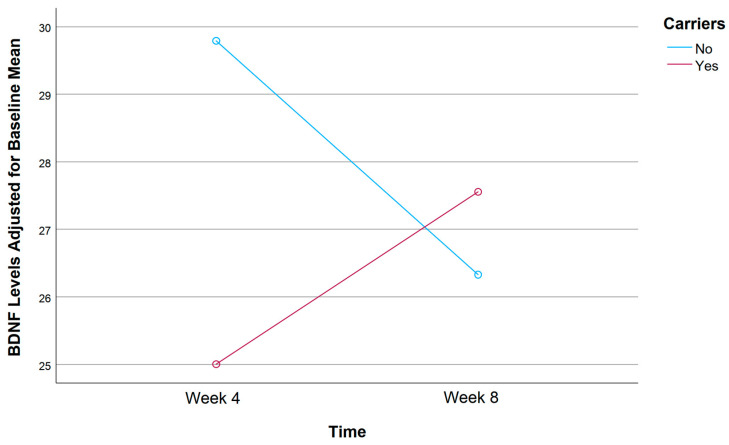
Effects of rs6265 A carrier status and time (Week 4 and Week 8) on BDNF levels.

A mixed ANOVA was conducted to evaluate the effects of rs6265 A carrier status and time on the adjusted mean BDNF levels from Week 4 to Week 8 (Figure 8). The entire cohort included non-carriers (n = 24) and carriers (n = 11) who had BDNF serum samples available at both follow-up time points. Non-carriers had an adjusted mean BDNF Wk4 of 29.79 and BDNF Wk8 of 26.33, indicating a decline in BDNF levels from Week 4 to Week 8. In contrast, carriers had an adjusted mean BDNF Wk4 of 25.00 and BDNF Wk8 of 27.56, showing an increase in BDNF levels over the same period. The difference between the groups did not reach statistical significance (F(1,32) = 2.890, *p* = 0.099).

### 3.3. rs10835210

**Figure 9:**
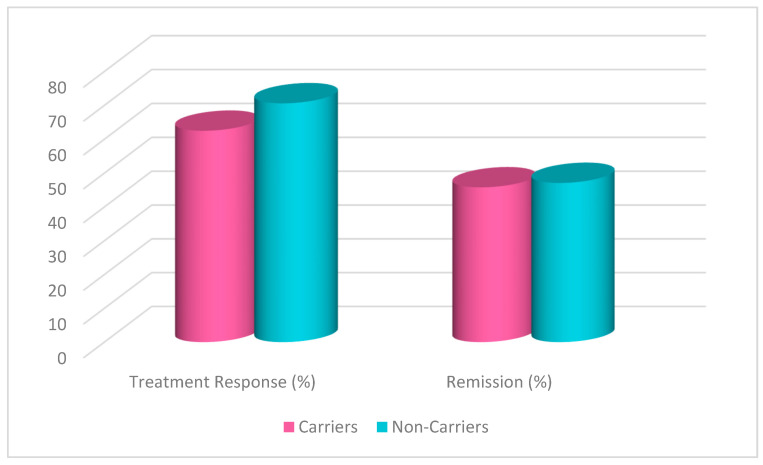
Effects of rs10835210 A carrier status on treatment response and remission.

A chi-square test was conducted to evaluate the effects of rs10835210 A carrier status on treatment response and remission (Figure 9) at Week 8. The cohort included non-carriers (n = 17) and carriers (n = 24). Non-carriers had a treatment response rate of 70.6%, while carriers had a response rate of 62.5%. The difference between the groups was not statistically significant (χ^2^(1) = 0.290, *p* = 0.591). Moreover, non-carriers had a remission rate of 47.1%, while carriers had a rate of 45.8%. The difference between the groups was not statistically significant (χ^2^(1) = 0.006, *p* = 0.938).

**Figure 10:**
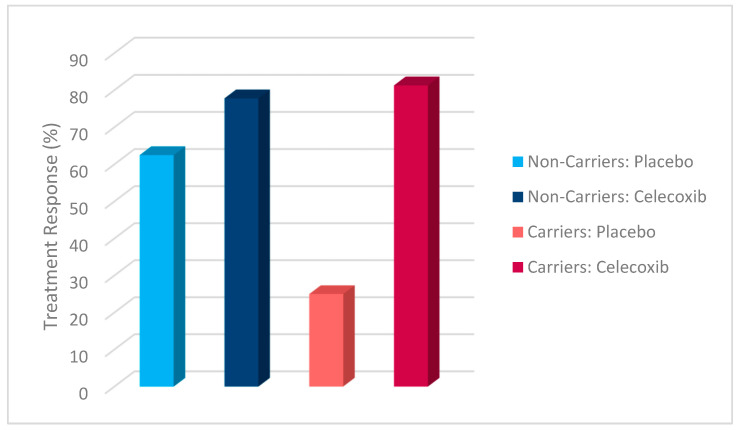
Effects of rs10835210 A carrier status and treatment group on treatment response.

**Figure 11:**
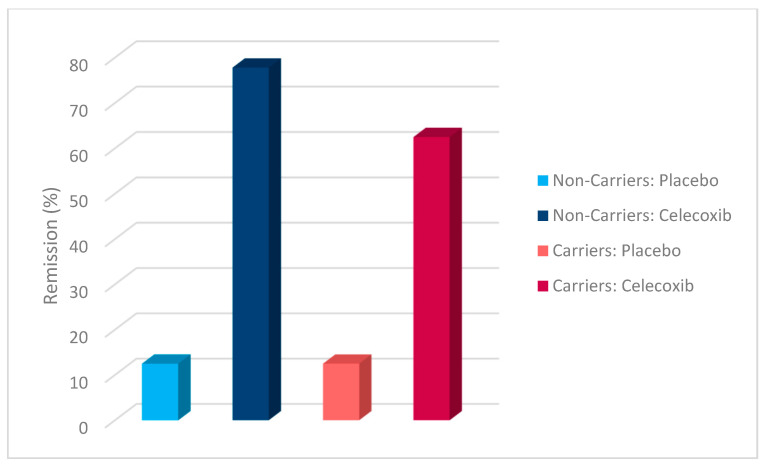
Effects of rs10835210 A carrier status and treatment group on remission.

A chi-square test was conducted to evaluate the effects of rs10835210 A carrier status and treatment group on treatment response (Figure 10) and remission (Figure 11). The cohort included non-carriers (n = 17) and carriers (n = 24). Among non-carriers, 62.5% of the placebo group and 77.8% of the celecoxib group responded to treatment. For carriers, 25% of the placebo group and 81.3% of the celecoxib group responded. The difference between the groups was not statistically significant for non-carriers (χ^2^(1) = 0.476, *p* = 0.490) but was statistically significant for carriers (χ^2^(1) = 7.200, *p* = 0.007).

Additionally, the effects of rs10835210 A carrier status and treatment group on remission rates demonstrated significant findings. Among non-carriers, 12.5% of the placebo group and 77.8% of the celecoxib group experienced remission. For carriers, 12.5% of the placebo group and 62.5% of the celecoxib group experienced remission. The difference between the groups was statistically significant for non-carriers (χ^2^(1) = 7.244, *p* = 0.007) and for carriers (χ^2^(1) = 5.371, *p* = 0.020).

**Figure 12:**
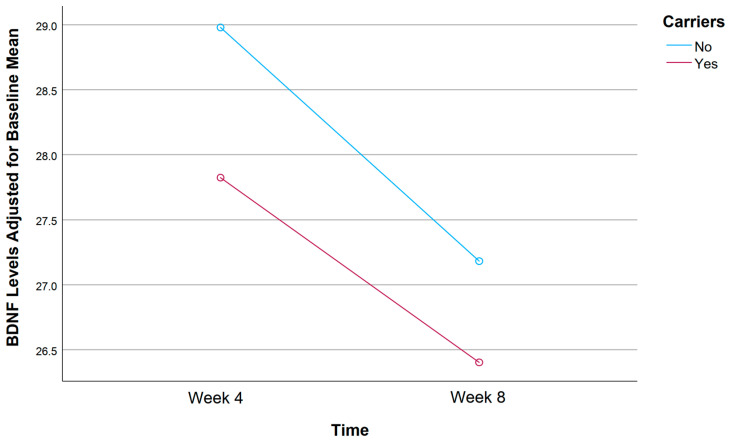
Effects of rs10835210 A carrier status and time (Week 4 and Week 8) on BDNF levels.

A mixed ANOVA was conducted to evaluate the effects of rs10835210 A carrier status and time on the adjusted mean BDNF levels from Week 4 to Week 8 (Figure 12). The entire cohort included individuals classified as non-carriers (n = 14) and carriers (n = 21) who provided BDNF serum samples at both follow ups. Non-carriers had an adjusted mean BDNF Wk4 of 28.98 and BDNF Wk8 of 27.18, while carriers had an adjusted mean BDNF Wk4 of 27.82 and BDNF Wk8 of 26.40. Notably, both groups demonstrated a slight decline in BDNF levels from Week 4 to Week 8, but non-carriers had slightly higher BDNF levels than carriers at both time points. However, the difference between the groups did not reach statistical significance (F(1,32) = 0.011, *p* = 0.917).

## 4. Discussion

The nervous and immune systems engage in complex communication regulated by multiple mechanisms, with peripheral cytokine activity and neuroinflammation contributing to the development of neuropsychiatric conditions [41]. Peripheral immune activation induces neuroinflammation, causing molecular changes in prefrontal cortex neurons that disrupt the excitatory and inhibitory balance and may underlie inflammation-related disturbances in behavior and cognition [42].

Neuroinflammation is associated with increased COX activity, with the COX-2 isoform playing a significant role in the central nervous system (CNS) [20]. Although the promoter-regulatory region of the PTGS2 gene encoding COX-2 is not typically active, it can be strongly and rapidly induced under specific conditions by growth factors and pro-inflammatory mediators [43]. Interestingly, COX-2 is also constitutively expressed in the CNS, particularly in glutamatergic neurons of the cortex and hippocampus, where its expression has been shown to be N-methyl-D-aspartate (NMDA) receptor-dependent. Spontaneous glutamatergic synaptic activity regulates this constitutive expression via stimulatory protein-1 (Sp1)- and cAMP response element-binding protein (CREB)-dependent transcriptional mechanisms [43].

The dysregulation of glutamate due to cytokine activity can cause excitotoxicity, reducing BDNF production, which is critical for neuronal health, neuroplasticity, and neurogenesis [18]. Our previous research identified an inverse relationship between perceived stress and BDNF levels [44], as well as significantly lower baseline serum BDNF levels in patients with TRBDD compared to healthy controls [45].

In the brain, COX-2 acts as a physiological regulator, but its pathological upregulation can produce reactive oxygen species and toxic prostaglandin metabolites, contributing to neuronal damage and neurodegeneration [46,47]. Celecoxib has demonstrated the ability to reverse memory deficits, likely by downregulating hippocampal COX-2 expression and upregulating the BDNF-TrkB signaling pathway [48]. In addition, inhibiting COX-2 may provide anxiolytic benefits for individuals with mood and anxiety disorders [49]. Moreover, by modulating COX-2 activity, celecoxib may influence indoleamine 2,3-dioxygenase, an enzyme involved in tryptophan and kynurenine metabolism that impacts 5-HT levels [26].

The current study aimed to determine whether genetic variations in the BDNF and BDNF-AS genes influence serum BDNF levels and their impact on treatment outcomes with escitalopram, either as a monotherapy or in combination with celecoxib. Our findings emphasize the significance of genetic polymorphisms in TRBDD, as treatment outcomes varied significantly between carriers and non-carriers of rs1519480G, rs6265A, and rs10835210A.

rs1519480 has been significantly associated with bipolar disorder [37,50]. It is an intronic variant in the BDNF-AS gene [51] and is in highly conserved regions, suggesting that it may play an important functional role [37]. Our findings indicate that ESC+CBX led to higher remission rates compared to ESC+PBO in both rs1519480G carriers (*p* = 0.022) and non-carriers (*p* = 0.009), with a particularly strong effect in non-carriers, where remission was 75% in the celecoxib group and 0% in the placebo group. These results suggest that celecoxib can significantly improve remission outcomes in both non-carriers and carriers, although it appears to have an even greater impact on G allele non-carriers in the treatment of patients with TRBDD.

The BDNF rs6265 polymorphism (Val66Met), a missense variant in the BDNF gene, results in a valine (Val) to methionine (Met) substitution at codon 66. This alteration affects BDNF protein expression and influences critical processes, such as neuronal plasticity, neurogenesis, and synaptic connectivity, potentially disrupting cellular processing and trafficking [51,52]. Additionally, rs6265 is associated with a non-coding transcript variant in the BDNF-AS gene [51]. In the present study, rs6265A non-carriers showed a significantly higher response to ESC+CBX compared to ESC+PBO (*p* = 0.005). Furthermore, both rs6265A carriers (*p* = 0.036) and non-carriers (*p* = 0.006) demonstrated significant differences in remission rates, with ESC+CBX being more effective in achieving remission compared to ESC+PBO. These results suggest that celecoxib may significantly improve remission outcomes in both non-carriers and carriers, with a particularly stronger effect observed in rs6265 A allele carriers.

Previous studies have shown inconsistent findings regarding the Val66Met polymorphism and its impact on antidepressant response. While some studies found no significant association between this polymorphism and the response to treatment or remission, including with escitalopram [53,54,55], other studies reported differing outcomes. For instance, Alexopoulos et al. (2010) found higher remission rates in Met carriers compared to Val/Val homozygotes after 12 weeks of escitalopram treatment [56]. Likewise, El-Hage et al. (2015) observed significantly better treatment responses in Met carriers and greater treatment resistance to escitalopram in Val/Val homozygotes following three weeks of treatment in inpatients with severe depression [10].

The rs10835210 polymorphism is associated with intron variants in both the BDNF and BDNF-AS genes [51] and has been linked to bipolar disorder [40] as well as escitalopram response [57]. The minor allele A of rs10835210 is associated with increased BDNF-AS mRNA expression in the frontal cortex, which inhibits BDNF transcription and reduces endogenous BDNF protein levels [58,59]. In our study, rs10835210A carriers demonstrated a significantly higher treatment response with ESC+CBX compared to ESC+PBO (*p* = 0.007), suggesting that celecoxib may be particularly effective in enhancing treatment response in this subgroup. Significant findings were also observed in terms of remission rates. rs10835210A non-carriers receiving ESC+CBX showed a much higher remission rate compared to those receiving ESC+PBO (*p* = 0.007), while carriers also exhibited significantly better remission outcomes with ESC+CBX compared to ESC+PBO (*p* = 0.020). These results indicate that celecoxib may play a crucial role in improving remission rates in both rs10835210A carriers and non-carriers, with a more pronounced trend observed in non-carriers.

This study provides valuable findings but has several limitations. First, the short duration of eight weeks may not have fully captured the long-term effects of treatment or changes in BDNF levels over time. Second, the relatively small sample size may have limited the power to detect significant differences in some comparisons, which could affect the generalizability of the findings to larger populations.

Additionally, in this study, BDNF levels were measured in serum. A common question among researchers is which medium is most appropriate for measuring BDNF levels [60]. High concentrations of BDNF are found in brain regions such as the hippocampus, amygdala, cerebellum, and cerebral cortices, with hippocampal neurons having the highest levels. A saturable transport system facilitates the transfer of intact BDNF across the blood–brain barrier [61]. Human platelets contain large amounts of BDNF protein, actively absorbed from the circulating BDNF pool [60,61]. Variations in serum BDNF concentrations among individuals are influenced by the activation-dependent release of BDNF from platelets [62]. Once released into circulation, BDNF has a half-life of less than 10 min and is primarily cleared by the liver, whereas platelets have a lifespan of 9 to 11 days. Thus, serum BDNF reflects cumulative activity over the preceding 10 days, while plasma captures more acute changes [63].

Moreover, the focus on three specific SNPs does not account for additional genetic variations that may influence BDNF levels and treatment efficacy. The interaction effects of BDNF SNPs with other genes and SNPs were not examined in this study, although synergistic genetic influences may play a role in treatment outcomes. For instance, polymorphisms in cytochrome P450 (CYP) enzymes, which mediate Phase I drug metabolism, are significant contributors to variability in enzymatic activity and interindividual differences in drug metabolism and therapeutic efficacy [64]. Specifically, escitalopram is primarily metabolized by CYP2C19 and CYP3A4, with a minor role for CYP2D6. The CYP2C19 and CYP2D6 genes are highly polymorphic, resulting in significant variability in enzymatic activity, while variations in CYP3A4 are less common [64,65]. Additionally, celecoxib is metabolized by CYP2C9 and CYP3A4 but also binds to and inhibits CYP2D6 without being metabolized by it [66]. The CYP2C9 gene is highly polymorphic, with two common variants, rs1799853 and rs1057910, associated with reduced CYP2C9 activity and altered celecoxib pharmacokinetics [67,68]. Furthermore, the role of CYP2D6 in celecoxib metabolism is variable and depends on CYP2C9 genotypes [69].

Finally, lifestyle factors such as exercise, diet, stress levels, and sleep quality can significantly influence BDNF levels, adding complexity to genetic associations and treatment effects. Intensive exercise increases BDNF serum levels compared to resting states, with levels peaking after 20 min of activity and returning to baseline within 10 min of recovery [70]. Notably, high-intensity interval training results in greater BDNF elevation than continuous exercise [70]. Adopting Mediterranean or ketogenic diets and using omega-3 polyunsaturated fatty acid supplements are also associated with higher circulating BDNF levels [71]. Additionally, intermittent fasting promotes BDNF signaling and an adaptive stress response [72]. In contrast, sleep disturbances, particularly insomnia, significantly lower BDNF serum concentrations [73]. Poor sleep quality raises stress levels, which suppresses BDNF secretion and increases vulnerability to depression [74].

Future studies should include larger and more diverse cohorts to enhance generalizability. Longitudinal designs with extended follow-up periods could help clarify the long-term effects of genetic polymorphisms on treatment outcomes and BDNF levels. Furthermore, expanding the range of genetic markers investigated may reveal additional factors influencing treatment response and remission, contributing to the advancement of personalized therapeutic approaches.

## 5. Conclusions

This study assessed the impact of three BDNF/BDNF-AS gene polymorphisms (rs1519480, rs6265, and rs10835210) on treatment outcomes and serum BDNF levels in patients with TRBDD over an eight-week period. Non-carriers of the rs6265 A allele and carriers of the rs10835210 A allele showed a significantly higher response to treatment with adjunctive celecoxib compared to escitalopram alone. Additionally, response rates to adjunctive celecoxib were significantly improved in both carriers and non-carriers across all three SNPs compared to escitalopram alone. However, remission rates were notably higher in non-carriers of the rs1519480 G allele and rs10835210 A allele, as well as in carriers of the rs6265 A allele. These findings suggest that adjunctive celecoxib enhances overall treatment outcomes, and the patient’s specific genetic profile plays a critical role in determining treatment response and the magnitude of remission.

## Data Availability

Data are contained within the article.
